# Brain SERT Expression of Male Rats Is Reduced by Aging and Increased by Testosterone Restitution

**DOI:** 10.1155/2013/201909

**Published:** 2013-12-18

**Authors:** José Jaime Herrera-Pérez, Alonso Fernández-Guasti, Lucía Martínez-Mota

**Affiliations:** ^1^Farmacología Conductual, Dirección de Investigaciones en Neurociencias, Instituto Nacional de Psiquiatría Ramón de la Fuente Muñiz, 14370 México City, DF, Mexico; ^2^Departamento de Farmacobiología, Centro de Investigación y de Estudios Avanzados del IPN, 14330 México City, DF, Mexico

## Abstract

In preclinical and clinical studies aging has been associated with a deteriorated response to antidepressant treatment. We hypothesize that such impairment is explained by an age-related decrease in brain serotonin transporter (SERT) expression associated with low testosterone (T) levels. The objectives of this study were to establish (1) if brain SERT expression is reduced by aging and (2) if the SERT expression in middle-aged rats is increased by T-restitution. Intact young rats (3–5 months) and gonad-intact middle-aged rats with or without T-restitution were used. The identification of the brain SERT expression was done by immunofluorescence in prefrontal cortex, lateral septum, hippocampus, and raphe nuclei. An age-dependent reduction of SERT expression was observed in all brain regions examined, while T-restitution recovered the SERT expression only in the dorsal raphe of middle-aged rats. This last action seems relevant since dorsal raphe plays an important role in the antidepressant action of selective serotonin reuptake inhibitors. All data suggest that this mechanism accounts for the T-replacement usefulness to improve the response to antidepressants in the aged population.

## 1. Introduction

Clinical studies propose a delayed response of aged patients to antidepressants as compared to young ones [1, 2]. Accordingly, we recently found in the chronic mild stress paradigm that middle-aged male rats (MA, 13–15 months) responded slower than young adults to the antidepressant treatment with citalopram (a selective serotonin reuptake inhibitor—SSRI—) [3]. The serotonin transporter (SERT) is the primary target of SSRIs and has a polymorphism in the promoter region of its gene with two variants: long (*l*) and short (*s*), interestingly, the *s*-variant has been associated to a reduced SERT expression and low serotonin uptake [4–7]. Patients carrying the *s*-variant (associated to low SERT expression) displayed a retarded response to SSRIs [8–10], suggesting a relationship between therapeutic response and number of SERTs [9, 11, 12]. On the other hand, it has been shown that in aged subjects there is deterioration of serotoninergic fibers in the rat forebrain [13] and reduced binding of [^11^C](+)McN5652 to SERT in several brain areas of *Rhesus* monkey, such as prefrontal cortex and hippocampus [14], a structure involved in the response to antidepressants [15]. On these bases we hypothesize that the impaired antidepressant-like response of MA rats to citalopram [3] is associated with an age-related reduction of brain SERT expression.

The mechanisms underlying the decreased SERT expression in MA subjects may include the reduction in testicular hormones found in these animals [16], which importantly affect the antidepressants' actions. Thus, in young animals, orchidectomy cancels the antidepressant-like effect of desipramine and fluoxetine [17], an effect recovered after testosterone (T) or estradiol (product of T-metabolism) replacement [17, 18]. In agreement, clinical studies showed that T-restitution ameliorated intractable depression [19] and restored the antidepressant effect of SSRIs in patients with major depression refractory to treatment [20]. In addition, in young rats, orchidectomy reduced the SERT mRNA expression in the dorsal raphe and the density of SERT binding sites in higher brain centers, effects that were prevented by T-treatment [21, 22]. These evidences support our second hypothesis: the age-related reduction of brain SERT expression is reversed by T-restitution of MA animals.

On these bases, the objectives of this study were to evaluate whether there is an age-dependent reduction of brain SERT expression and to determine if T-restitution to gonad-intact MA rats recovers the SERT expression. Thus, we quantified the brain SERT immunoreactivity in brain areas involved in depression and/or antidepressants' response [23–25]: prefrontal cortex, lateral septum, hippocampus, and raphe nuclei.

## 2. Material and Methods

### 2.1. Animals

Five young (3–5 months) and eight MA (13–15 months) male Wistar rats, obtained from the vivarium of the *Instituto Nacional de Psiquiatría Ramón de la Fuente Muñiz *(INPRFM), were located in jumbo acrylic cages (4-5 per cage) and maintained on a 12 : 12 light-dark inverted cycle (lights on at 10:00 h) under controlled temperature and humidity, with water and food available *ad libitum*. Animal management was done according to the general principles of laboratory animal care (NIH publication 85-23, 1985). Experimental procedures were performed according to the Mexican official norm for animal care and handling (NOM-062-ZOO-1999) and approved by the Ethical Committee of the CINVESTAV-IPN and INPRFM. All efforts were made to minimize the number of animals used and their suffering.

### 2.2. Testosterone Restitution of Middle-Aged Rats

To restore the T levels of gonad-intact MA animals (1.02 ± 0.35 ng/mL) to those of young adults (3.97 ± 1.09 ng/mL) [16], we subcutaneously placed individual T-containing pellets in the cervical region of rats under tribromoethanol (200 mg/kg, Sigma-Aldrich) anesthesia, as previously described [26]. The pellets were polydimethyl silicone tubes (ID/OD: 1.57 mm/3.18 mm, Dow Corning) of 1 cm long, filled with T-propionate (~9 mg, Sigma) and sealed with pure silicone. After restitution, animals were left undisturbed for 3 weeks before brain extraction; at this time T levels were 4.67 ± 0.81 ng/mL [26]. Restitution was done in gonad-intact MA rats in order to simulate the hormone restitution done in men with partial androgen deficiency.

### 2.3. Histology

Young adults (*n* = 5) and MA rats with (*n* = 4) or without (*n* = 4) T-restitution were anesthetized with ketamine (100 mg/kg, i.p.; Pisa)/xilazine (20 mg/kg, i.p.; Bayer) and perfused through the left ventricle with phosphate buffered saline solution (PBS: NaCl, 0.13 M; NaH_2_PO_4_, 0.003 M; Na_2_HPO_4_, 0.007 M; pH 7.2) containing heparin (1 mL/l, Pisa), followed by a 4% paraformaldehyde (Sigma) solution in PBS. Brains were extracted, cryoprotected in a PBS solution containing 30% of sucrose (Sigma) and 0.1% of thimerosal (Sigma), and stored at 4°C. Brains were sectioned in coronal slices of 40 *μ*m thick using a cryostat (Microm HM505N) and stored at 4°C in 30% sucrose solution in PBS. For immunofluorescence, four adjacent brain slices containing prefrontal cortex (Bregma 3.14 mm), lateral septum (Bregma −0.24 mm), hippocampus (Bregma −3.24 mm and −4.80 mm), or raphe nuclei (Bregma −7.56 mm) were selected. Identification of brain areas was done according to Paxinos and Watson rat brain atlas [27].

### 2.4. Immunofluorescence

Brain slices were washed with PBS solution and incubated at room temperature (RT), shaking for 2 hours in solution A: PBS containing goat serum (10%, Sigma), bovine serum albumin (BSA, 1%, Research Organics), and triton TX-100 (0.3%, Sigma). Slices were washed with solution B: PBS containing triton TX-100 (0.15%) and incubated on a shaker with the primary antibody (mouse anti-rat SERT, Chemicon International) diluted 1 : 500 in solution A as follows: one hour at RT, all night at 4°C, and again for one hour at RT. Slices were washed with solution B and incubated at RT on a shaker for two hours with the secondary antibody (goat anti-mouse IgG labeled with Oregon Green, Invitrogen) diluted 1 : 100 in a PBS solution containing goat serum (5%) and triton TX-100 (0.3%). Slices were washed with solution B and mounted on slides using Prolong Antifade kit (Invitrogen) and coverslips. Negative controls were processed as described above, but primary antibody was omitted.

### 2.5. SERT Immunoreactivity Analysis

Immunofluorescence was observed with a 40X oil immersion objective (S Fluor, NA 1.3, Nikon) in an inverted microscope (Nikon, Diaphot 300) equipped with an epifluorescence system (excitation filter: 480 ± 15 nm; dichroic mirror: 505 nm; emission filter: 535 ± 20 nm, Nikon), coupled to a Xenon arc lamp (75 W). SERT immunoreactivity (IR) images were digitalized with a cooled digital CCD camera (ORCA-ERC4742-95). For each image, a frequency histogram of fluorescence intensity was generated; in this histogram, a threshold was established to eliminate unspecific fluorescence. The threshold (mean + 3 standard deviations) of the non-T-treated MA animals (samples with the highest threshold) was used to eliminate unspecific fluorescence in all preparations. Once eliminated this background fluorescence, the number of pixels with specific mark, was quantified and expressed as percentage relative to total pixels in the analyzed area (relative SERT-IR); this parameter was considered a semiquantitative method to determine SERT expression. This process has been previously used by other authors to detect changes in various markers after immunoreactive techniques, including SERT [28–30].

Image acquisition and SERT-IR quantification were done using Metafluor software, version 4.0 (Universal Imaging Corporation). SERT-IR was quantified bilaterally in the following specific sites: prefrontal cortex (PC): cingulated, prelimbic, infralimbic; lateral septum (LS): dorsal, intermediate, ventral; hippocampus (HIP): dentate gyrus, CA1, CA2, CA3; raphe nuclei (RN): dorsal (dorsal and ventral portion) and median.

### 2.6. Statistics

Comparison of relative SERT-IR for each brain area in all experimental groups was done using a one-way analysis of variance (ANOVA), followed by the Student Newman Keuls (SNK) as the *post hoc* test. Student's *t*-test was used when required. Statistics was carried out using the Sigma Plot software, version 11. A value of *P* ≤ 0.05 was considered as statistically significant.

## 3. Results

**Figure 1:**
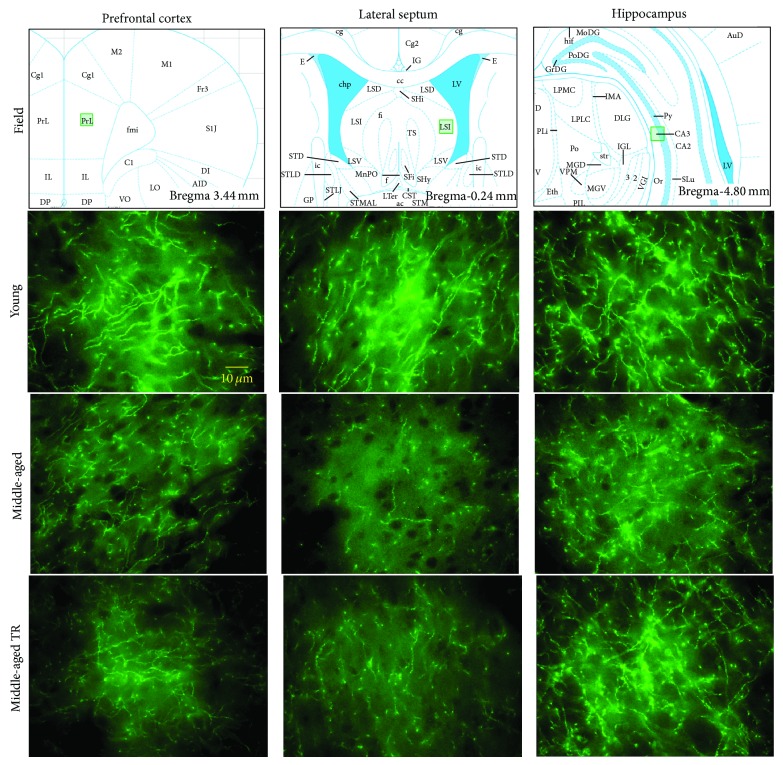
Photomicrographs of SERT-IR in prefrontal cortex, lateral septum, and hippocampus of young adults and middle-aged rats without or with T-restitution (TR). The field of analysis is indicated.

**Figure 2:**
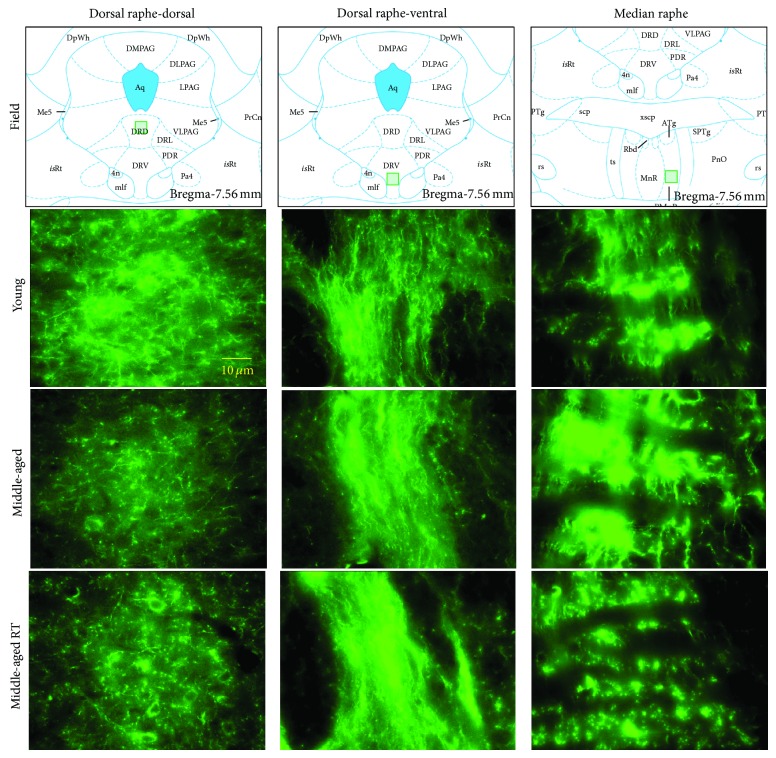
Photomicrographs of SERT-IR in dorsal and median raphe nuclei of young adults and middle-aged rats without or with T restitution (TR). The field of analysis is indicated.

Figures 1 and 2 show representative photomicrographs of SERT-IR in several brain areas of young adults and MA rats with or without T-restitution. The immunofluorescence was observed as punctuate fibers with highly labeled varicosities forming complex meshworks. The highest SERT-IR was found in the dorsal (ventral portion) and median RN (Figure 2). In the ventral portion of dorsal RN, bundles of labeled fibers projecting dorsoventrally were appreciated, whereas in the median RN the bundles appeared to project caudofrontally. Finally, in the dorsal portion of dorsal RN labeled cellular bodies were found (Figure 2). Immunodetection of SERT was considered specific, because in experiments where the primary antibody was omitted, immunofluorescence was not observed (data not shown).

Fibers with SERT-IR in young animals were larger, more robust, and better defined (e.g., HIP, Figure 1) than in MA rats, except in the dorsal (ventral portion) and median RN (Figure 2). We also observed a lower incidence of labeled cell bodies in dorsal RN of MA animals (Figure 2) as compared to young ones.

The pattern of SERT-IR in T-treated MA rats was similar to the other groups. However, SERT-IR fibers in this group were better defined in some brain areas (e.g., HIP: Figure 1 and dorsal RN: Figure 2) as compared to MA animals without T-restitution.

**Figure 3:**
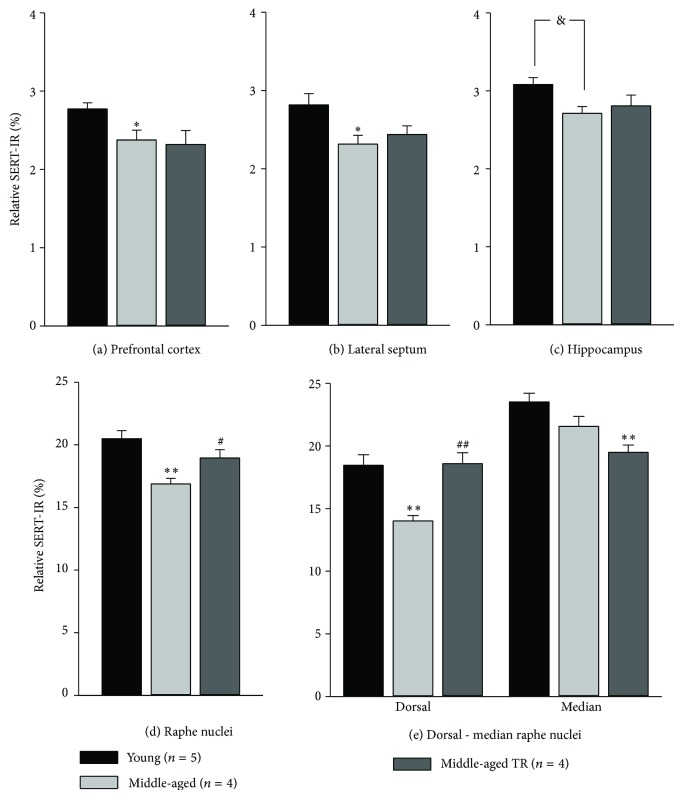
Relative SERT-IR in the analyzed brain regions. Data are expressed as mean ± SEM. SNK test: ^*^ *P* < 0.05, ^**^ *P* < 0.01 versus young group; ^#^ *P* < 0.05 versus middle-aged group without T restitution (TR). Student's *t*-test: ^&^ *P* < 0.05 versus young group.

Analysis of brain SERT-IR in all groups is shown in Figure 3. One-way ANOVAs indicated differences of relative SERT-IR in PC (*F*_2,10_ = 4.087, *P* = 0.05; Figure 3(a)) and LS (*F*_2,10_ = 4.353, *P* = 0.044; Figure 3(b)). SNK test indicated that MA rats without T-restitution displayed lower SERT expression than young animals in these areas (*P* < 0.05), while the transporter expression in T-treated MA rats tended to be lower with respect to young adults (PC, *P* = 0.065 and LS, *P* = 0.062). The one-way ANOVA for relative SERT-IR in the HIP (Figure 3(c)) indicated a tendency for differences between the groups (*F*_2,10_ = 3.566, *P* = 0.068); thus, analyzing only for age, the paired comparison of relative SERT-IR between young adults and MA rats without T-restitution indicated a significant difference (*P* = 0.022; Student's *t*-test).

Regarding the RN (Figure 3(d)), the one-way ANOVA evidenced differences in relative SERT-IR between the groups (*F*_2,10_ = 9.292, *P* = 0.005); SNK test indicated that SERT expression in young adults and T-treated MA rats was similar, while the transporter expression in these groups was higher than in MA rats without T (*P* = 0.004, *P* = 0.042, resp.). Because T-restitution reversed the effect of aging in RN, we further analyzed differences in this structure. Figure 3(e) shows the relative SERT-IR in dorsal and median RN. The one-way ANOVA indicated differences in the dorsal region (*F*_2,10_ = 10.869, *P* = 0.003), where the SERT expression was lower in MA rats without T-restitution as compared with young adults (*P* = 0.002) or T-treated MA males (*P* = 0.006). Meanwhile, in the median RN the differences (*F*_2,10_ = 8.484, *P* = 0.007) were determined by the higher SERT expression in young adults as compared with T-treated MA rats (*P* = 0.006), but no differences were found between MA animals with or without T-restitution.

## 4. Discussion

The morphology of the SERT-IR fibers found in this study were similar to those previously described for the male rat's brain, including the presence of immunolabeled cell bodies in the dorsal RN [31]. The presence of SERT-IR in these neurons is also in agreement with *in situ* hybridization [32, 33] and immunocytochemical [34] studies.

### 4.1. Age-Dependent Reduction of Brain SERT Expression

The age-dependent reduction in brain SERT expression reported here agrees with previous works conducted in *Rhesus* monkeys [14] and humans [35, 36]. In view that SERT indicates serotoninergic innervation [31, 37–39], we suggest that the reduced SERT expression in MA animals represents a deterioration of the serotoninergic system. This notion is supported by the aberrant serotoninergic fibers found in the brain of aged rats [13], the reduced binding potential of [^11^C](+)McN5652 to brain SERT in aged humans [36], and the low brain serotonin levels associated with aging [40]. These data, together with the idea that the antidepressant effect of SSRIs depends on the blockage of an adequate number of SERTs [9, 14], support the hypothesis that the low SERT expression in MA animals accounts for their retarded response to citalopram [3]. Thus, in MA animals, citalopram blocks fewer SERTs than in young ones, inducing lower increases in serotonin levels in the synaptic cleft; thus, compared to young animals, serotoninergic synapses of MA rats require a longer exposition to SSRIs to reach the serotonin levels needed to trigger the antidepressant-like effect. In agreement, clinical studies show that a higher brain SERT availability predicts a better response to SSRIs [11], and that patients with the *s*-allele of SERT gene show a retarded response to SSRIs [8–10]. The reduced number and intensity of immunolabeled fibers in the brain of MA rats reported here, together with a poor neural arborization of PC [41] or HIP [42, 43], low neurogenesis [44], and reduced BDNF expression in HIP [45], indicate an age-related deterioration of neural plasticity. According to the net theory of antidepressant action, the antidepressant treatment reestablishes the communication in the neural circuitry of depressed subjects [46], implying that the antidepressant response requires adequate brain plasticity mechanisms [15, 47]. Thus, the reduced neural plasticity of aged animals [44, 45] contributes to their poor response to antidepressants.

### 4.2. T-Restitution Increases SERT Expression Only in Dorsal Raphe of Middle-Aged Rats

The increased SERT expression in the dorsal RN of MA animals after T-restitution agrees with a study done in young rats where the orchidectomy-dependent reduction of SERT mRNA expression in RN was prevented by T-administration [22]. However, contrasting with the current study, Fink's group showed that T-treatment also increased the SERT expression in the forebrain of orchidectomized young adult rats [21]. The differences could be explained by methodological dissimilarities such as SERT-measurement technique, age variations, time of treatment, restitution method, and hormone status at the time of T-restitution.

The T-dependent SERT expression increase in the RN of MA rats may facilitate SSRIs' actions, since this region controls the firing rate of serotonergic neurons [48, 49]. Thus, the antidepressant response to citalopram in T-treated MA rats could be triggered in a shorter time as compared with MA animals without T-treatment. Alternatively, T could facilitate the antidepressant response by increasing BDNF levels and hippocampal neurogenesis [44, 45], a process implied in antidepressant response [15]. These ideas are supported by basic studies where gonadal hormones restored the antidepressant-like effect of fluoxetine in orchidectomized young rats [17], and by clinical studies showing that T-supplementation improved the antidepressant action of SSRIs in hypogonadal patients with major depression refractory to treatment [20].

### 4.3. Limitation of the Study

One factor that could limit the conclusions derived from this study is the number (4 or 5) of animals used per group; however, the correspondence of our results with previous studies from several laboratories (as discussed above) gives support to our findings.

## 5. Conclusion

The current study demonstrates a reduced SERT expression in PC, LS, HIP, and RN of MA rats, which may be related with the retarded response of these animals to antidepressants. T-restitution to MA rats increased SERT expression in the dorsal RN, an important site for the therapeutic action of SSRIs, suggesting that this hormone would improve the response to SSRIs in experimental and clinical conditions.
